# “*Are we in this together?*”: embedding social identity detection in drones improves emergency coordination

**DOI:** 10.3389/fpsyg.2023.1146056

**Published:** 2023-09-07

**Authors:** Anastasia Kordoni, Carlos Gavidia-Calderon, Mark Levine, Amel Bennaceur, Bashar Nuseibeh

**Affiliations:** ^1^Department of Psychology, Lancaster University, Lancaster, United Kingdom; ^2^School of Computing and Communications, The Open University, Milton Keynes, United Kingdom; ^3^Lero – the Science Foundation Ireland Research Centre for Software, University of Limerick, Limerick, Ireland

**Keywords:** social identity, emergency, rescue drone, sociotechnical, decision making

## Abstract

Autonomous systems, such as drones, are critical for emergency mitigation, management, and recovery. They provide situational awareness and deliver communication services which effectively guide emergency responders’ decision making. This combination of technology and people comprises a socio-technical system. Yet, focusing on the use of drone technology as a solely operational tool, underplays its potential to enhance coordination between the different agents involved in mass emergencies, both human and non-human. This paper proposes a new methodological approach that capitalizes on social identity principles to enable this coordination in an evacuation operation. In the proposed approach, an adaptive drone uses sensor data to infer the group membership of the survivors it encounters during the operation. A corpus of 200 interactions of survivors’ talk during real-life emergencies was computationally classified as being indicative of a shared identity or personal/no identity. This classification model, then, informed a game-theoretic model of human-robot interactions. Bayesian Nash Equilibrium analysis determined the predicted behavior for the human agent and the strategy that the drone needs to adopt to help with survivor evacuation. Using linguistic and synthetic data, we show that the identity-adaptive architecture outperformed two non-adaptive architectures in the number of successful evacuations. The identity-adaptive drone can infer which victims are likely to be helped by survivors and where help from emergency teams is needed. This facilitates effective coordination and adaptive performance. This study shows decision-making can be an emergent capacity that arises from the interactions of both human and non-human agents in a socio-technical system.

## Introduction

Technologies with the capacity to act autonomously, such as drones, have become integral to various emergency operations. They have been deployed in the aftermath of major incidents, such as the 9/11 attacks (Murphy et al., 2016), hurricane Katrina (Pratt et al., 2009), and the floods in Mexico in 2016 (Jiménez-Jiménez et al., 2020). Their deployment in such large-scale emergencies has proven useful for emergency responders who need to engage with multiple, complex tasks (Zaccaro et al., 2012), and coordinate activities under time pressure, physical and psychological pressures (Power and Alison, 2017a) to reduce harm and safe lives (Brown et al., 2021). Drones can help in the different tasks of the response phase in these large-scale incidents by providing situational awareness (Erdelj et al., 2017), navigating spaces inaccessible to people (Falanga et al., 2018), transporting medical and urgent supplies (Liu and You, 2020), and facilitating the search and rescue of stranded people (Van Tilburg, 2017).

Researchers working on improving the contribution of drones in emergency response have mostly focused on situational awareness—that is, the capacity to make sense of the context as accurately as possible. There is comparatively little research on using drones to *interact* with the different groups of people and other technologies in an emergency response. In the few cases where drones were designed to do some kind of interaction, the interaction was limited to the drone and the emergency responder (i.e., drone operator; Doherty et al., 2016). However, successful responding to a major incident requires the coordination and cooperation of multiple groups of human (emergency responders, affected individuals) and non-human (drones, telecommunications) agents. In this paper, we utilize recent work in social psychology on how group processes, specifically the concept of a shared identity (see Drury, 2018 for a review), can be harnessed to improve a specific facet of the response in large-scale emergencies, the evacuation of injured individuals. More specifically, we investigate whether and how a “shared identity” can be operationalized in an autonomous system, such as a drone. We, then, examine the consequences of this identity-informed drone for the evacuation effort. In doing so, we expect an identity informed drone to contribute to better help allocation, and by implication, better distributed decision making among the agents involved, both human and non-human. Thus, the present research seeks to address two questions:

RQ1: How can social identities be computationally detected, classified, and incorporated in a rescue drone?
RQ2: Can an identity-adaptive rescue drone demonstrate greater effectiveness in a rescue operation than a non-adaptive rescue drone?

To address these questions, we employed a methodological approach that uses linguistic features to detect and classify indicators of social identity during the emergency and leverages this information to inform the decision making of an adaptive drone. Specifically, our model accounts for *what survivors say* during an emergency to infer the type of identity upon which they act. Based on this information, the identity-informed drone can decide whether a victim is likely to be helped by fellow survivors or whether assistance from emergency responders is needed, and, thus, what action needs to be taken. In the following, we provide an overview of the theoretical and practical drivers of our investigation and describe the architecture developed in previous research (Gavidia-Calderon et al., 2022), which allows for identity adaptation. We, then, describe our proposed methodology for this adaptation based on survivors’ linguistic features. We evaluate our identity-informed autonomous agent using linguistic and synthetic data in an evacuation scenario of an injured individual.

### Human-drone interaction and decision making in emergencies

Research on (semi)-automated rescue systems has provided evidence that rescue drones can reduce an emergency team’s cognitive load during an emergency response. Rescue drones reduce response time (Tanzi et al., 2016), provide more precise and insightful information in real time, such as of the type of incident, its magnitude, additional hazards, the number and location of injured people and possible access and evacuation routes (Chuang et al., 2019), due to advanced sensing capabilities. In this way, they help increasing a sense of being in control of the situation (Subramaniam et al., 2012) and ensure that emergency commanders refrain from endangering rescuers (Karaca et al., 2018), especially in cases of mass shootings, fires, potential toxic exposure, or explosives (Abrahamsen, 2015). These rescue drones may also include capabilities to detect, assess and control cognitive load using physiological data (Dell’Agnola et al., 2020). In other words, they can reduce *endogenous uncertainty*—the difficulty in decision making due to uncertainty associated with ambiguous information and risks that prevent the decision maker from developing enough situational awareness to project an outcome (Shortland et al., 2020).

Less research has focused on how rescue drones can help reduce the uncertainty for the actions of the public during an operation (van den Heuvel et al., 2012). This source of endogenous uncertainty has been associated with concerns about the actions of people affected by the emergency as well as the difficulty of protecting people in the risk area while ensuring the protection of people outside the risk area (Power and Alison, 2017b). This type of uncertainty has been found to result in decision inertia—a cognitive process of decision derail due to redundant deliberation on the complexity of the problem (Alison et al., 2015)—and the “least-worst” type of decision making (Power and Alison, 2017a,b). To the best of our knowledge there is no research which explores how drone systems might reduce the endogenous uncertainty which results from how ordinary people might behave in large-scale emergencies.

Leveraging the role of survivors in coordinating evacuation activities in large scale emergencies is advantageous for different reasons. In these incidents, survivors tend to outnumber first responders. Since they are already in the field, they tend to provide medical assistance, scene management, food, water, and help with evacuations (Rapaport and Ashkenazi, 2021), even before the emergency response teams arrive. Indeed, research has shown that panic rarely occurred during emergencies (Johnson, 1988; Kuligowski and Kuligowski, 2009; Drury, 2018). Instead, survivors tended to behave orderly and in a cooperative manner (Rodriguez et al., 2006; Drury et al., 2009a; Drury and Tekin Guven, 2020). Recent evidence from CCTV footage of real-life emergencies corroborated that during a train evacuation, survivors exhibited helping behaviors and coordinated activities (Philpot and Levine, 2022). When demand outstrips resources, and dynamic changes, uncertainty and time limitations may have irreversible effects, survivors play an active role in coordinating an emergency response by providing support to fellow survivors.

Survivors are also more likely to trust a rescue robot^1^ to guide them to safety (Wagner and Robinette, 2015). In evacuation scenarios, survivors tended to follow rescue robots in the nearest exit (Jiang et al., 2019; Zhou et al., 2022). This safety behavior was corroborated in lab-based experiments (Robinette et al., 2016), agent-based simulation models of evacuations (Sakour and Hu, 2016) and virtual reality evacuation scenarios (Wagner and Robinette, 2015). It was more prevalent in high-risk emergency situations and even when the rescue robot was witnessed to making a mistake before the evacuation operation (Robinette et al., 2016; however, see Lee and See, 2004, for potential pitfalls of over-trusting automation). Notably, the level of adherence to the robot’s guidance was determined by the safety behaviors of the other individuals in the scene. Inconsistency between survivors’ behavior and robot’s guidance, namely individuals moving in a different direction than the direction recommended by the robot, decreased the probabilities that the individual would follow the robot (Nayyar and Wagner, 2019). By contrast, consistency between survivors’ behavior and the robot’s guidance encouraged adherence to the robot’s guidance and tended to organize a crowd into groups to orderly split across all possible exits (Zhou et al., 2022). Thus, there is an existing level of trust and cooperation between survivors and between survivors and rescue robots in evacuation efforts.

The present research capitalizes on the active role of the so-called “zero responders”—survivors who help in emergencies—, and especially the psychological drivers of their behavior, namely the salient social identity they act upon. By detecting survivors’ identity via the use of language and adapting to it, a rescue drone can become the intermediary that manages the complexity of decision making in an evacuation operation. This is achieved by balancing the socio-cognitive (Gore et al., 2018a) and socio-technical aspects of the emergency response. By detecting the survivors’ identity, the rescue drone contributes to a better coordination of evacuations between survivors and first response teams. This capacity can improve the evacuation effort by mobilizing civilians based on adapting to their social identity status in large-scale incidents. It can also help first responders’ decision making by creating a shared understanding of behavior and activities between zero responders, first responders, and the autonomous agent, and by providing a basis to prioritize certain rescues over others in a high-risk, high-stake, challenging environment. Instead of treating an autonomous agent as a tool to merely establish communication during an operation (Gutiérrez-Reina et al., 2018), our research explores the capacity of an autonomous system to be a strategic actor that reconciles relationships and coordinates actions through a *socio-technical process*.

### A social identity approach to emergency responses

Theoretically, our research is driven by social identity principles (e.g., Spears, 2021). A social identity is the part of an individual’s self-concept that derives from their knowledge of belonging to a social group(s) along with the values and emotional significance attached to this membership (Tajfel, 1978, p.63). As a psychological concept, social identity has practical value for societal issues and interventions, including in emergency settings. A shared group membership was evident among survivors in the response phase of large-scale emergencies, such as terrorist attacks (e.g., Drury, 2018), outdoor music events of 1,000 people turning into an emergency (Drury et al., 2015), earthquakes (Vezzali et al., 2015), floods (Ntontis et al., 2017), public subway train evacuations (Philpot and Levine, 2022), and evacuations of large buildings in fire (Tekin and Drury, 2021).

This research has led to the *social identity model of collective psychosocial resilience* (Williams and Drury, 2009, 2010; Drury et al., 2009b). This model suggests that during an emergency, the affected individuals turn from a physical crowd into a psychological crowd due to their common experience (common threat) of the emergency. In this way, a collective self-categorization is developed that is perceived to better fit their social reality. This elicits a sense of common fate (Turner et al., 1987), which becomes the basis for a “we-ness” among survivors, a new shared identity that overrides other possible self-categorizations (Clark, 2002; Drury et al., 2009a; Drury, 2012). This process leads to a cognitive and relational transformation, being characterized as a shift in one’s goals from self-interest to collective interest, and acts of solidarity, respectively (Drury, 2012). Survivors sharing an identity, display helping behaviors toward other survivors, expect to be supported, and cooperate to achieve common goals (Levine et al., 2005). In this way, acting upon a shared identity in emergencies corresponds with the idea of a “social cure” (Jetten et al., 2012) because it facilitates pro-social outcomes and emotional support. However, adopting a shared identity is not universal, in the sense that there is a minority of survivors who tend to lack this sense of unity and, thus, they act to save themselves (Drury et al., 2009a). As such, it would be advantageous for a rescue drone to be able to detect what kinds of identities are salient because these identities tend to elicit different behaviors (e.g., helping a victim vs. saving oneself).

Conceptually, our research proceeds from the assumption that a rescue drone that can detect whether a survivor is currently in the state of shared identity, will improve the evacuation effort. This means that by inferring a person’s salient psychological identity, the adaptive robot can more accurately decide which action to perform, to ask for help from the survivor, or to call a first responder. In this work, we suggest that the robot could infer a shared or not shared identity from *natural language*. Given that a shared identity can be manifested through language (Melucci, 1995), it is expected that by detecting *what survivors say* during an emergency operation, an identity adaptive drone will infer a survivor’s probability of acting in line with a shared identity or not.

### Using natural language as an identity indicator

Language and identity are interconnected and dependent on the context in which they appear (Bucholtz and Hall, 2005). People are active communicators of their social identities (Klein et al., 2007). Depending on the social groups to which they belong, they tend to adapt the way they talk about ingroup and outgroup members (Porter et al., 2016). Using language that emphasizes membership ties and promotes social bonding around a common threat has been found to reflect the salience of a shared identity (Hardy et al., 2005). People tend to communicate a sense of affiliation to indicate a shared identity (Penner, 2002). This is reflected in the use of inclusive language (e.g., Gulliver et al., 2021) and especially the use of “we” pronouns (Gonzales et al., 2010) that exemplify a sense of community (Vambheim et al., 2013). This evidence suggests that linguistic information can be a robust indicator of a shared identity (Jaspal, 2009) that can be used for group classification (Koschate et al., 2021).

The relationship between language and evacuation behavior has predominantly focused on the effectiveness of linguistic cues as a means of communication during an evacuation. Proulx et al. (2001) suggested that people tend to perceive non-linguistic cues, such as alarms, as more ambiguous and less effective compared to linguistic cues. The spoken language of the survivors, the linguistic cues used by the rescue technology and egress time are interconnected (Meacham, 1999) and determine survivors’ movements and evacuation outcomes (Mazur et al., 2019). Nevertheless, using linguistic cues to infer group memberships in evacuations has been underexplored. Previous attempts have examined other types of behavioral data as indicators of a shared identity in evacuations. Those included physical proximity—how close survivors are—(Philpot and Levine, 2022); speed of movement—how fast they move in the area—(von Sivers et al., 2016); walking style—patterns of walking behavior—(Templeton et al., 2018); and facial expressions (Kantharaju and Pelachaud, 2021). To the best of our knowledge, this is the first study that seeks to do this through analysis of natural language.

### An architecture of an adaptive drone

**Figure 1:**
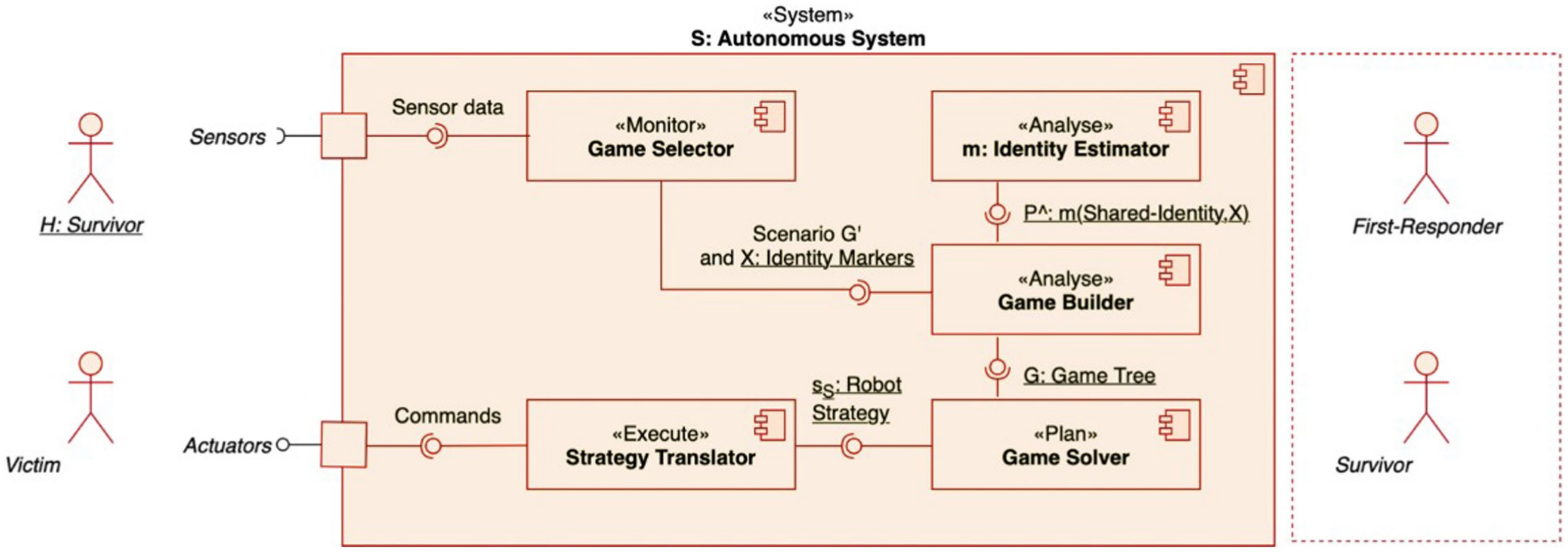
*MAPE-K* framework for the identity-adaptive autonomous agent. Component diagram of our adaptive architecture: the game selector triggers an identity-driven scenario *G*^′^ that there is an injured individual close to another survivor and sends identity marker information *X (linguistic data)* to the game builder. This component assembles the game tree *G* incorporating the identity probabilities *P*^ˆ^ produced by the identity estimator. The game solver calculates the robot strategy *sS* that the strategy translator transforms into actionable commands.

To be able to accurately detect and make decisions in line with a survivor’s identity, the autonomous system needs a self-adaptive architecture that includes an identity classifier-a function to computationally detect an identity and quantify it to inform decision making. Such an architecture was designed in previous work (Gavidia-Calderon et al., 2022) using a *MAPE-K* framework for self-adaptive systems (Kephart and Chess, 2004). Under this framework, the autonomous system’s components were organized within four elements: *Monitor, Analyze, Plan, and Execute* that operate over a shared *Knowledge*. The *MAPE-K* framework associated with this work is presented in Figure 1.

The *Monitor* element collects data from the sensors of a drone to support two functions of the *Analyze* element: to assess whether identity-adaptation is needed and to collect the information that is needed to complete the models within the *Knowledge* element. Whether identity adaptation is needed, is determined by the presence or absence of a first responder close to a victim (an injured individual). If the drone detects a first responder close to the victim, adaptation for providing help is not required. If the drone detects another survivor close to the victim, adaptation is required (Gavidia-Calderon et al., 2022).

The kind of adaptation that is required depends on the salient identity of the detected survivor and its probability. If the survivor acts upon a shared identity, they are more likely to help the victim. If they act upon a personal identity, they are less likely to help the victim. If a shared identity is detected, the drone can ask the survivor to help the victim evacuate. If a personal identity is detected, the drone needs to request help from a first responder remotely (hence, the adaptation). In Gavidia-Calderon et al. (2022), these interactions between a victim who requires help, a survivor in close proximity, and the drone are represented using Game Theory. Game theoretic models are models that represent interactions of conflict or cooperation between rational decision makers who adopt certain strategies to perceive an impact, i.e., maximize their payoff (Tadelis, 2013). Under these rationality assumptions, it is predicted that only a subset of outcomes is possible because a decision maker’s adopted strategies, such as the strategies of the rescue drone, are expected to be the best responses to the strategies of another decision maker, such as the survivor’s strategies. These optimal outcomes are called Nash equilibria and tend to be stable, as any kind of deviation leads to payoff loss. Each identity adaptation has a game theoretic model that drives its decision making. However, in our evacuation scenario, this game theoretic model is incomplete in the sense that there is uncertainty about the survivor’s adopted identity which is the determinant of the drone’s decision. Bayesian game theoretic models can model this uncertainty based on known information, such as identity indicators, which then predicts another set of optimal outcomes, named Bayes Nash equilibria. These Bayesian game theoretic models constitute the shared *Knowledge* of the architecture. As such, each identity adaptation has a Bayesian game theoretic model that drives its decision making. In this paper, we focus on the model that uses identity indicators to provide identity probabilities in a single scenario of a victim being helped by either a fellow survivor or a first responder with a single game model.

To inform the Bayesian game model regarding the type of identity adoption, the *Monitor* element searches for and collects identity-related sensor data. Then, this data are conveyed to the *Analyze* element which includes an identity classifier.^2^ This classifier calculates the probability of the survivor to hold a shared identity or not. These probabilities inform the Bayesian game theoretic model of the *Knowledge* element. Then, the *Plan* element calculates the Bayesian Nash equilibria, namely a predicted strategy of the survivor and the response that the drone should have to this strategy. The *Execute* element translates these strategies into robot commands, i.e., asking for help from the survivor or the first responder (see Gavidia-Calderon et al., 2022 for a more detailed explanation of the identity-adaptive architecture that was adopted here). The focus of this paper is on our methodological approach for the identity classifier of this architecture based on linguistic data, which is presented next.

## Methods

### Building the identity classifier: data collection and coding procedure

The current research has been granted ethical approval by the FST Research Ethics Committee (FSTREC), Lancaster University. Data were collected from YouTube videos in the public domain based on interviews of survivors of real-life emergencies who described their experiences in the news, documentaries, talk shows, or event anniversaries. We used the transcript function of YouTube.^3^

When people describe an experience, they tend to use expressions such as “I said (…) and then, they said (…),” “we heard somebody saying…,” and which can make the experience psychologically present (Rubin et al., 2003). We identified what people said after these expressions (i.e., actual words spoken) in the transcripts and collected these expressions. Data were collected from 66 YouTube videos. We collected expressions that occurred during an interaction that a survivor reported having with a fellow survivor and was relevant to their evacuation experience, namely what they reported saying to each other in the aftermath of the event and during the evacuation effort. This included language aiming to inform of the situation or cause some kind of action. These expressions were collected from videos referred to major incidents, such as evacuations after terrorist attacks, large building fires, and mass shootings that took place in the last 25 years all over the world. Data collection resulted in a corpus of 200 English expressions of what survivors reported saying during the emergency. For our model, the utterance level was taken to be statements with complete meaning during each reported interaction. To ensure that identity dynamics could be captured in a contextualized manner, utterances included one-person statements (up to three sentences long), or a short dialogue between two survivors (see, for example, Ahmadvand et al., 2019 for contextual dialogue and transfer learning).

Given the nature of the data, coding a survivor’s expressions relative to the identity dimension can be seen as a text classification problem. To assess whether these expressions were indicative of a shared identity or not, two independent raters coded the extracted expressions with two categories: (1) reflects a shared identity and (2) does not reflect a shared identity. The criteria for this coding were common for the two raters and derived from the *social identity model of collective psychosocial resilience* (Williams and Drury, 2009, 2010; Drury et al., 2009b), and research on the linguistic features of identity (e.g., Gulliver et al., 2021). Examples of the extracted data for each one of the criteria are presented below.

An extracted expression was considered to be indicative of a shared identity when:

1. They are indicative of “we-ness,” namely when they show affiliation. Linguistically this is associated with words, such as “we” and “us” and represent a set of actions with a shared meaning (Boyd et al., 2022). For example, “We have to go. We’ve got to go now!”
2. They reflect collective coordination of actions and support. This is related to expressions that show people’s interaction to coordinate actions to safety, namely expressions of mutually joining an act of helping each other (Ntontis et al., 2017). This criterion reflects an “I do this, you do that, so we can both be safe” way of talking. For example, “–Bite it out. Try again. Can do it. Is it attached to a piece of wood? – Yes. –The nail is going to come off. Try again. I’ll catch you on the other side.”
3. They reflect emotional support and empowerment. This is indicated by expressions of social bonding, where people tend to share or amplify common identity categories (e.g., families), which has been found to promote trust (Drury et al., 2019). For example, “You have to think about your family. Got to do it.” Empowerment is associated with encouragement- a “do not give up” way of acting toward fellow survivors (Pütz et al., 2011). For example, “I will meet you later.”

By contrast, an extracted expression was considered to *not* be indicative of a shared identity when:

1. It reflects a personal identity. The focus here is on the use of the “I” pronoun and expressions associated with one’s saving themselves regardless of what other fellow survivors do or whether they need help. For example, “I am getting heck out of here!”
2. It is a declaration statement. This is an expression where people tend to declare what is happening but there is no indication of identity. For example, “A bomb has gone off!”

Manual labeling was preferred over a more automated approach, such as using the LIWC software (Boyd et al., 2022). Although LIWC includes dictionaries for positive and negative emotions, identity affiliation, and words associated with pro-social behavior, it cannot adequately capture activity coordination and support, as well as empowerment in a contextualized manner. At the time of data collection, automatic labeling of empowerment processes was found to be conducted by text classifiers that were trained on manually labeled sub-samples that were not English and not relevant to the emergency context (e.g., Verberne et al., 2019). To ensure that all nuances associated with the context and the identity criteria are adequately represented in our dataset, and for reasons of consistency, we proceeded with a manual labeling approach.

To assess the reliability of the raters’ codes, we calculated the Gwet’s AC reliability coefficients, which shows the interrater agreement and the degree of this agreement (Gwet, 2014). This statistical test is more robust than Kappa in rare event situations and able to handle categorical data (Gwet, 2016). Thus, it was considered to be a better fit to our data. The agreement coefficient was 0.81, *SE* = 0.04, 95%CI [0.73, 0.89], which shows adequate reliability (Landis and Koch, 1977). To ensure that no bias was introduced in the model due to the raters’ coding, those expressions that the raters disagreed upon were excluded from analysis (*n* = 19). The final dataset included 181 extracted expressions coded as indicative of a shared identity or not shared identity, for which there was agreement between the raters. The data, labeling, and the classifier code are provided at https://github.com/cptanalatriste/transformer-type-estimator.

### Building the identity classifier: training and validation

From a corpus of 181 labeled expressions, we assigned 126 expressions (70%) for training and validating the classifier, and 54 (30%) for evaluating evacuation performance. Due to the relatively small size of the training dataset, we used a pre-trained transformer model that allows for minimal training data (Huggins et al., 2021). Specifically, we used a pre-trained BERT model (Devlin et al., 2018), which stands for Bidirectional Encoder Representations from Transformers. The BERT model computes numerical representations of natural language, using a transformer encoder over text instances. For the identity classifier, we used a HuggingFace’s open-source implementation of the BERT model (Tunstall et al., 2022), which was pre-trained on the Book Corpus dataset (Zhu et al., 2015) and the English Wikipedia. This model requires textual input to be processed with WordPiece, a subword tokenization algorithm (Schuster and Nakajima, 2012).

**Figure 2:**
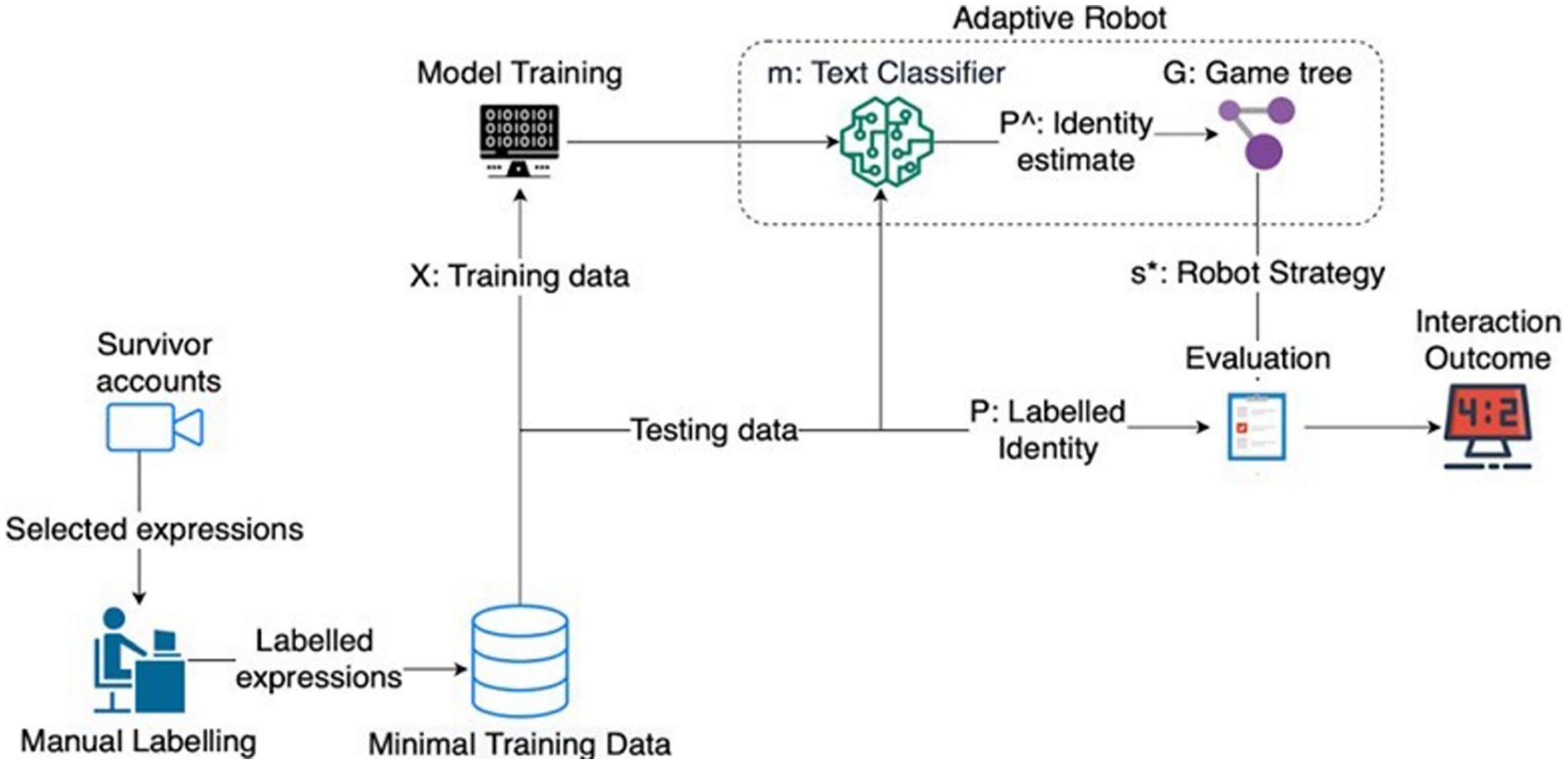
Classification process and expected use in adaptive autonomous systems.

To adjust the pre-trained BERT model to reflect our classification problem, namely whether an expression is associated with a shared identity or not, we used a fine-tuning process, as follows. First, we appended a classification layer to the pre-trained BERT model in order to support identity inference. Second, we trained this extended model using our new dataset (survivors’ expressions) by taking the pre-trained BERT parameters as a starting point. This type of modeling was conducted in Python. From the 126 labeled expressions, we used 63 (50%) for the training pipeline and the rest for validation and probability calibration. When engaging in the supported adaptation-driven scenario, this modified model can receive the survivor’s expressions as input. The output is the probability that the survivor has adopted a shared identity or not. Then, these probabilities can inform the game theoretic models of an identity-adaptive drone (in line with the *Knowledge* element). This process is depicted in Figure 2.

As shown in Figure 2, we manually labeled expressions extracted from survivors’ testimonies as indicative (or not) of a shared identity. Part of this data was used for training (x) and validating the identity classifier (m), and the rest as test data for evaluation. The identity classifier produces the probabilities of the inferred identity (P^), which inform the game model (G). The game model, then, calculates the optimal strategy (s*) for the drone and the ground-truth identity of the labeled testing expression (P) that are used for performance evaluation and, therefore, action to be taken. The advantage of this approach is that the processing of linguistic data and strategy calculation happens at run-time (see Gavidia-Calderon et al., 2022). As events unfold, identity dynamics may change in the evacuation scenario, which can be reflected in the language used. At run-time, this linguistic change imposes a change in the probabilities of inferred identity. Since the game theoretic models can run in multiple iterations until the situation resolves (help is provided), this change in identity probability would drive a change in the strategies adopted by the drone (whether to ask for help from the survivor or remotely call for first response aid). Theoretically, this adaptive approach is more likely to meet changes imposed by contextual demands of an evacuation, such as when a survivor with a shared identity find themselves unable to help the victim due to disruption.

## Results

### Building the identity classifier: performance accuracy

To test whether social identity can be, indeed, computationally classified to inform the decision making of a rescue drone (RQ1), the overall performance of the identity classifier was estimated through the Area Under the ROC Curve (ROC AUC), which is recommended when aiming to report prediction accuracy for categorical variables (e.g., Kosinski et al., 2016). AUC shows how well the classifier separated between an expression being indicative of a shared identity or not. An AUC score of 0.50 is equivalent to guessing, while an AUC score of 1 is perfect classification.

We trained the identity classifier over the training dataset (*n* = 63), using the validation dataset (*n* = 63) for parameter tuning (batch size, epochs, and learning rate) and probability calibration. For further details regarding the training process, our code is freely available at GitHub.^4^ After training and probability calibration, the ROC AUC was 0.89 over the validation dataset. This result shows that the identity classifier could sufficiently distinguish between the two identity types stemming from survivors’ reported sayings during an evacuation.

### Evaluation based on linguistic data

To test whether an autonomous agent that incorporates this identity classifier can indeed contribute to an improved evacuation response (RQ2), we compared the performance of our identity-adaptive architecture based on language against two non-adaptive architectures, namely architectures that did not account for identity probabilities to calculate the best strategy in the game models: (i) a pro-self-oriented drone that, regardless of shared identity probabilities, assumes a survivor never helps a victim in close proximity, and thus, it always calls for first-responder support, and (ii) a pro-social oriented drone that, regardless of shared identity probabilities, assumes a survivor always helps a victim in close proximity, and thus, it always asks for survivor’s help to evacuate.

For this evaluation, we assumed that each expression from the testing dataset (*n* = 54) corresponds with one interaction, between the rescue robot, the survivor using the expression, and the victim in close proximity, namely the condition that requires identity adaptation to facilitate the rescue effort. This means we accounted each extracted expression as a different scenario running 54 times. Building on the game theoretic models of the *Knowledge* element proposed by Gavidia-Calderon et al. (2022), from the robot’s perspective, the best outcome is to receive help from the survivor evacuating the victim when it detects that the survivor is likely acting upon a shared identity. In this way, we would expect three evacuations: the survivor and the victim’s evacuation, and another victim’s evacuation who could be helped by the first responder elsewhere (since the first responders wasn’t called here). The worst outcome, under a shared identity assumption, is the survivor ignoring the victim. Using this game-theoretic model, we calculated the expected number of evacuees for the identity-adaptive and the non-adaptive architectures from the payoff the drone would obtain given its strategy in every game-theoretic model (to ask for help from the survivor or the first responder) and the ground-truth identity of the labeled testing expression.

Over 54 victim-survivor interactions on the test data, the identity-adaptive architecture has a payoff of 149 expected evacuations, which is better than 138.8 expected evacuations of the pro-social architecture and 145.4 expected evacuations of the pro-self-architecture. These results suggest that the identity-adaptive architecture based on linguistic features tends to outperform the non-adaptive architectures. Although the performance gain seems to be modest, this preliminary result suggests the viability of our approach and speaks to its potential applications.

### Evaluation based on synthetic data

While the evaluation of the identity adaptive robot using linguistic data showed that this robot is likely to improve the expected number of successful evacuations, this result was driven by modeling one interaction between a victim, a survivor, and the identity adaptive robot per labeled expression in the test dataset. However, a real emergency situation includes more than one interactions between these agents. At the same time, survivors tend to adopt a shared identity, but the degree of identity adoption may vary. To further illustrate the capacity of an identity adaptive robot to improve the number of expected evacuations, we explored its performance in these conditions (Gavidia-Calderon et al., 2022).

Our goal was to calculate the expected number of evacuations, as in the previous section, on 30 scenarios composed by 33 victim-survivor-robot interactions. In this case, the expected number of evacuations was calculated for survivor populations that varied in their degree of a shared identity adoption, which was made to range from 50 to 80%. For this analysis, we relied on a synthetic data generation process that is detailed at: https://github.com/cptanalatriste/wdywfm-adaptive-robot.

As can be seen in Table 1, the results indicated that across all levels of a shared identity adoption, the average number of evacuations for the identity adaptive robot was larger compared to a pro-self and a prosocial robot. These findings suggest that an identity adaptive robot is likely to improve an emergency response by providing a better distribution of help between first responders and zero responders.

**Table 1:** Preliminary evaluation results based on synthetic data.

Robot	*r*_G_ = 50%	*r*_G_ = 60%	*r*_G_ = 70%	*r*_G_ = 80%
Adaptive	91.6	92.8	93.9	95.3
Pro-self	89.2	89.9	90.5	91.2
Pro-social	86.1	89.0	91.6	94.0

*r* represents the degree of shared identity adoption. *G* represents the game tree *G*. *r*_G_ represents the degree of identity adoption in the game theoretic model. Scores represent the average number of expected evacuations, as adopted by Gavidia-Calderon et al. (2022).

## Discussion

One of the key challenges for emergency response decision making systems is how they anticipate the behavior of ordinary people in a crisis (Power and Alison, 2017b). Understanding and adapting to group behaviors in an emergency is crucial for planning and coordinating an emergency response. In this paper, we investigated the capacity of a rescue drone to facilitate overcoming this major barrier to emergency responders’ decision making by detecting, classifying, and accounting for survivor’s salient identity.

Our findings support the potential of automatically detecting and inferring the salient identities of survivors using their spoken language during an evacuation scenario. We also show that an identity adaptive robot can help to improve coordination between survivors and first responders (as shown by an increase in the expected evacuations) by using this ability to detect shared identity. The present research contributes methodologically, empirically, and theoretically to understanding this kind of socio-technical coordination. In so doing, it contributes to the creation of an interdisciplinary space in which opportunities for integrating psychological research on group processes in emergencies and rescue robotics might be explored.

Methodologically, our research has employed a novel combination of psychologically informed natural language processing techniques with software engineering. This enabled us to investigate how psychological concepts such as one’s social identity can be detected, classified, and quantified to inform the decision making of an adaptive robot. An advantage of this methodology is that the probability for the survivor’s expected behavior derives from the psychological processes that frequently drive this behavior, therefore, providing a more accurate representation of survivors’ decision making.

Another methodological contribution is associated with the theoretically informed use of verbal interaction as an analytical point for text classification models. In doing so, our methodology can inform recent developments in modeling “conversational” agents. Our classification model accounts for the verbal interaction between a survivor and a victim to infer a shared identity. In tandem, rescue drones are fully equipped with advance communicative capabilities, such as audio transmitters, leading to a more direct interaction with human agents through language (Mayer et al., 2019). Albeit insufficient to assume a conversation between actors (drone, survivor, and victim) at this stage, our methodological approach is a first step toward unfolding interactions in contextual dialogue models (e.g., Ahmadvand et al., 2019) for rescue drones using psychological principles as their classification criteria.

Empirically, our research has presented novel evidence on the nature of human-robot interactions in crisis management, contributing to emerging work on the role of a shared understanding between human and non-human agents (Werkhoven et al., 2018). Instead of focusing solely on technical aspects that the robot understands its functionality and goals, and, thus, it expects a human agent to behave in line with its expectations (always saving oneself or always helping others), our findings suggest that the integration of identity principles may be capable of accounting for a more comprehensive set of human behavior patterns in emergencies, which, in turn, can improve people’s understanding of the autonomous agent’s decision making. Our results suggest that in multiple evacuation scenario iterations, an increase in expected evacuations is associated with this shared understanding between agents.

Theoretically, our paper contributes to the way we might conceptualize decision making in emergencies—and the way ‘decisions’ can be thought about in socio-technical systems. Instead of focusing on the emergency responder as the only decision maker, an identity adaptive rescue drone allows for decision distribution between human and non-human agents. Our work demonstrates the theoretical possibility of being able to train an autonomous system to make decisions about the behavior of humans using psychological theory about the way humans behave in emergencies. In this way, it opens up the theoretical space for a more sophisticated understanding of how humans and machines can work together in emergency response systems.

Regarding its applied implications, our proposed approach could benefit emergency responders by providing real-time information of help allocation. An identity adaptive drone detects indicators of survivors’ identity, such as their linguistic features in our study, to decide the state of a survivor’s identity. It, then, uses this information to provide an improved distribution of help between first responders and zero responders. Due to its adaptive nature, it can also inform when changes in help provisions need to be made. By mobilizing civilians based on adapting to their social identity, our approach could help first responders prioritize the evacuation of victims who have no other source of aid. In this way, an identity adaptive drone contributes to alleviating a main source of endogenous uncertainty and facilitates the planning, coordination, and decentralization of the operation, which tends to improve emergency responders’ cooperation with other partners and the public (Brown et al., 2021).

An additional advantage of this methodology is that it could potentially facilitate decision making in other organizational contexts. Given that (i) language embeds social categories, such as gender, age, or culture (e.g., Eastman, 1985; Peersman et al., 2011), (ii) robotics have found their way in our everyday life, and (iii) the game trees of the identity adaptive robot rely on human and non-human agents’ interaction, the identity adaptive robot could further support decision making for help distribution in medical assistance or task distribution, and team building among employees in an organization.

### Limitations and future research

This paper has attempted to develop a novel approach to investigating how social identity can be computationally detected, classified, and inform the decisions of an adaptive rescue robot, and demonstrated that this identity adaptive robot is likely to improve the number of successful evacuations. However, we need to acknowledge some limitations and highlight potential directions for future research.

The main psychological component that determines the decisions of the adaptive drone is social identity, as reflected in spoken language. While this has many advantages given that language is a dominant communication mechanism, in our approach the verbal detection and processing is English only. This limitation on the drone’s ability to detect other spoken languages could inadvertently increase the survival rate of some evacuees over others, such as non-English speakers (Wagner, 2021). Identity adaptive drones for emergencies need to be able to detect the spoken language of as diverse a population as possible. They also need to be able to identify other communication cues to provide equal opportunity for people with speech impediment. In our preliminary research, English was chosen because it is the common language among the investigators and because previous psycholinguistic investigation of identity was largely conducted in English. Follow-up research needs to address these limitations for future adaptive drones.

This latter point taps into another critique of the spoken expressions used to train and test our model. Although they derive from real-life emergencies and survivors, this association is indirect since our data were extracted by narratives of their emergency experience. Given the importance of storytelling and memory on these events (East et al., 2010), we treated these oral expressions as proxies of the actual verbal interactions. A further limitation of working with this type of data is that their external validity may be limited. Emergencies are unique in their circumstances, which makes it difficult to ascertain whether these specific verbal behaviors would be detected across different emergency situations. To mitigate this, we extracted oral expressions that were as generic as possible and from critical incidents globally. Accordingly, the use of synthetic data suggested that an identity-adaptive robot may be able to accommodate context-dependent differences relative to identity adoption, such as the type and severity of the emergency and number of people involved. Our findings showed that an increase in shared identity adoption is likely associated with an increase in the number of evacuations, suggesting that an attempt to increase shared identity adoption may help the evacuation effort as a whole. To detect nuances associated with specific contextual factors (e.g., Delmerico et al., 2019), individual differences in the communicative style among survivors and to expand on non-dyadic interactions (Schneiders et al., 2022), our future research aims to use other types of data, such as drone data, to directly explore identity detection and manifestation in different emergency socio-technical systems.

Another limitation is more generally associated with classification as a dominant supervised learning approach. Whereas for some models classification can be straightforward with the distinction between classes being clear, the classification of psychological concepts is trickier (see Alfano et al., 2022 for a similar discussion). For example, the conceptualization of a shared identity is theory informed. In this case, there is extensive research on the concept and researchers tend to agree on its individual linguistic components (e.g., Gulliver et al., 2021). What constitutes a “not shared identity” is not as clear as a shared identity. In our investigation, the class of “not shared identity” was informed by two components: theory and context. The social identity approach in emergencies has shown that although the majority of survivors tend to help, which reflects a salient shared identity, there is a minority of survivors who aim to save themselves, which reflects a salient individualistic (personal) identity (Drury, 2018). At the same time, much evidence in evacuations suggests that, especially at early times of the emergency, survivors tend to declare the situation. This kind of classification is not free from subjectivity and possible solutions are very challenging. One possible approach to tackle this may be by using a one-class classification, where the model is only trained by the class on which there is consensus (Fard et al., 2019). Given that our goal here was that of adaptiveness in decision making, exploring this alternative model in other applications of rescue drones, while ensuring robustness and security capabilities to protect fundamental human rights, such as privacy, is a fruitful direction for future research.

Despite these limitations, our work advances psychological modeling by showing how we can classify and include social identity ideas in rescue robot design, thus introducing a new approach for adapting rescue robots to group behaviors. Understanding how interaction opportunities between human and non-human agents can benefit from psychological properties of groups enables cooperation not only between people, but also between people and robots. This has clear implications for both emergency response and the developers of rescue robotics.

## Conclusion

This paper presented a novel approach that informs an adaptive rescue drone to make decisions for help allocation based on a survivor’s group identity. Our findings suggest that social identity detected in language can be a valuable means for the decision making of a rescue drone. By providing a proof-of-concept example on how to construct such a model and evaluate its quality, we provided initial evidence that this identity adaptive drone may play a strategic role in emergency responders’ decision making and adaptive performance. We believe that our approach is a step toward more resilient socio-technical systems for large-scale critical incidents.

## Data availability statement

The datasets presented in this study can be found in online repositories. The names of the repository/repositories and accession number(s) can be found in the article/supplementary material.

## Ethics statement

This study involving anonymized textual materials was reviewed and approved by the Faculty of Science and Technology Research Ethics Committee (FSTREC), Lancaster University, United Kingdom. Written informed consent was not required for this study in accordance with the national legislation, ethics guidelines for internet-mediated research, and the institutional requirements.

## Author contributions

AK and CG-C have made substantial contributions to the conception of the study, data collection, analysis, and interpretation of the research data, and in preparing the manuscript for publication. All authors contributed to the article and approved the submitted version. All authors contributed to the article and approved the submitted version.

## Funding

This work was supported by the Engineering and Physical Sciences Research Council (grant numbers EP/V026747/1 and EP/R013144/1); and Science Foundation Ireland (grant number 13/RC/2094_P2).

## Conflict of interest

The authors declare that the research was conducted in the absence of any commercial or financial relationships that could be construed as a potential conflict of interest.

## Publisher’s note

All claims expressed in this article are solely those of the authors and do not necessarily represent those of their affiliated organizations, or those of the publisher, the editors and the reviewers. Any product that may be evaluated in this article, or claim that may be made by its manufacturer, is not guaranteed or endorsed by the publisher.

## Footnotes

^1^In this paper, we define a drone as *a flying robot that can be remotely controlled or fly autonomously using software-controlled flight plans in its embedded systems along with different kinds of onboard sensors* (Lutkevich, 2021).

^2^This is represented with a Type estimator in Gavidia-Calderon et al. (2022).

^3^YouTube transcript service has shown the lowest Word Error rate (Kim et al., 2019). To validate this, two of the researchers independently watched the videos and extracted the expressions. Only those expressions for which both researchers and the YouTube transcripts were in full agreement were included in the dataset.

^4^ https://github.com/cptanalatriste/transformer-type-estimator
